# Characterization of the Effects of a Six-Month Dancing as Approach for Successful Aging

**DOI:** 10.1155/2019/2048391

**Published:** 2019-06-17

**Authors:** Maria Grazia Vaccaro, Giulia Izzo, Alessandro Ilacqua, Silvia Migliaccio, Carlo Baldari, Laura Guidetti, Andrea Lenzi, Aldo Quattrone, Antonio Aversa, Gian Pietro Emerenziani

**Affiliations:** ^1^Neuroscience Centre, “Magna Graecia” University, Catanzaro, Italy; ^2^Department of Experimental and Clinical Medicine, “Magna Graecia” University, Catanzaro, Italy; ^3^Department of Movement, Human and Health Sciences, “Foro Italico” University, Rome, Italy; ^4^University eCampus, Novedrate, Italy; ^5^Department of Experimental Medicine, “Sapienza” University, Rome, Italy

## Abstract

Aging is accompanied by a decline in multiple domains. Positive effects of dance practice on several health issues have been evaluated in young adults, while the effects of regular social dance practice on physical fitness, sexual health, and cognitive functions have not been studied yet in older experienced dancers. Thus, the aim of this study has been to investigate whether a 6-month social dance practice might influence fitness performance, sexual health, and specific cognitive functions and/or mood characteristics in older experienced dancers. Thirty experienced dancers (age: 71.2±5.1 years, 18 females/12 males) were enrolled from the dance school “NonSoloLiscio” of Catanzaro. Body composition, physical fitness, sexual health, and cognitive functions were assessed before (T0) and after (T6) intervention. After 6 months of dance practice, percent of fat mass (%FM) significantly decreased (p<0.01), while fat-free mass (FFM) significantly increased (p<0.01) in both genders. Moreover, significant main effects of time on physical fitness tests, such as chair stand test (CST) (p<0.01), gait speed (p<0.05), and timed up and go (p<0.05), were found. Sexual health was significantly higher in males than in females at T0 and no significant effects of dance on subjects' sexual health were found. Interestingly, trait of anxiety significant decreased (p<0.05) and perception of retrospective memory significantly increased (p=0.05) after training independently of gender. Our preliminary results suggest that, even in older intermediate-level dancer, the practice of social dance might positively influence body composition and also increase fitness performance, memory functions, and anxiety. In contrast, no effects on sexual health were observed after 6 months of dancing.

## 1. Introduction

Aging is accompanied by a decline in multiple domains, including physical fitness, psychological aspects, mood, social relation, and sexuality. Identifying the factors that underlie resiliency is essential for assessing a decline's risk for future deterioration and for designing interventions to promote healthy aging [1]. Recent data suggest that dementia affects approximately 24 million people globally, but, given the increasing number of older subjects, this number is expected to grow to approximately 81 million people by the year 2040 [2]. To contrast this trend, the National Institute for Health and Care Excellence recommends that adults increase their physical activity (PA) levels to reduce the risk of dementia [3, 4]. Behavioral activities have been suggested to be a potential tool for improving cognitive capacities in both healthy [5] and pathological populations [6]. Moreover, it is well known that PA might have positive effects on physical fitness [7, 8], physiological state [9], and cognitive functions [10, 11] in elderly individuals. Recent scientific evidences have shown that personality is an important aspect of sexual functioning and a good sexual health is a marker of positive well-being [12, 13]. In fact, it has been recently defined how the concept of well-being should also include sexual health, even in the later decades of life.

Sexuality is influenced by different aspects such as physiological, psychological, and social states [13]. For this reason, the study of sexuality in older adults requires multidisciplinary approaches that involve sexual attitudes and mental and physical fitness. Studies reported that elderly people with cognitive decline and dementia had fewer sexual activities than cognitively intact and nondemented persons [14, 15]. Moreover, scientific evidence [16] showed that there is a strict relationship between active sexuality, preserved cognitive function, thyroid function, and appropriate physical fitness in elderly subjects. Cognitive impairment is characterized by problems in thinking, memory, language, and judgment that are greater than cognitive changes in normal aging [17]. Physical activities differ considerably in the level of sensorimotor complexity, cognitive demand, and degree of social interaction, and so, the reduction of cognitive decline might also be dependent on the type of exercise performed [18]. Some hypotheses have been proposed, including that aerobic exercise improves insulin sensitivity and glucose control, which may decrease the incidence of amyloid plaque creation leading to Alzheimer's disease [19]. Thus, dance might be proposed as a possible nonpharmacological intervention for promoting mental and physical well-being in seniors [20] and could improve short-term memory and executive functioning in old-old people [21]. Moreover, the use of traditional Greek dance could improve static and dynamic balance control in the elderly [22].

Dance consists of complex elements, such as synchronization of movement to music, memorization of step sequence, and social interaction which, on its own, is recognized as having a beneficial effect on cognition [8, 17]. It requires associations of several cognitive and physical functions from perception, execution, memory, and motor skills [23]. Indeed, over the last decade, dance is gaining popularity as a therapeutic activity for improving the cognitive skill of older people affected by Parkinson's disease [24, 25] and other pathologies. Song [26] demonstrated the positive effects of dance-based movement training on physical and psychological functions in subjects affected by severe cerebellar ataxia. Furthermore, any dance style can induce positive functional adaptations in elderly, above all related to both balance [27] and muscle strength [28]. However, these scientific evidences were focused on the effects of dance in beginner dancers or nonhealthy subjects. Therefore, for promoting a successful aging, it would be interesting to analyse the effects of dance not only in beginner, but also in intermediate dancers. Indeed, regarding physical performance, the positive effects of dance evaluated in beginner dancers might be attenuated after a long time of practice. Moreover, despite these positive effects of dance on physical fitness and cognitive functions, no studies were conducted on the effects of dance on sexuality in elderly. Therefore, the aim of our study was to analyse the effects of 6-month social dance practice on physical fitness, sexual health, cognitive function, and subjective perception of memory deterioration in older intermediate-level dancer.

## 2. Methods

### 2.1. Participants

Thirty active older subjects (age: 71.2±5.1 years, 18 females and 12 males) were recruited in this study from the Dance School “NonSoloLiscio” of Catanzaro. All participants gave their written informed consent before inclusion in the study. Five participants dropped out during the study (16%). Therefore, the final number of participants was twenty-five. The causes of dropout were new development of disease and/or pains not related to the dance program. Subjects did not receive any financial incentives. This study complied with the Declaration of Helsinki and gained ethical approval by the “*Magna Graecia*” University of Catanzaro Ethical Committee (EudraCT protocol no. 2016-005198-11). Inclusion criteria consisted in age >65 years and at least five years of dance practice. Exclusion criteria were as follows: physical impairment and neuropathy, severe psychological, psychiatric, and neurological diseases, uncontrolled cardiovascular diseases, and hyperglycaemia. Before the intervention, all subjects had practiced dance at least two times a week for five years. All subjects were intermediate-level dancers, and no one was engaged in any dance competition.

To exclude any thyroid disease, blood samples were drawn in the morning after an overnight fast in all subjects. Thyroid-stimulating hormone (TSH), free triiodothyronine (FT3), and free thyroxine (FT4) were assayed. Then, after a clinical examination, subjects underwent physical fitness, sexual health, and cognitive assessments.

### 2.2. Experimental Intervention

Participants carried out their dance practice 4 days a week for 6 months. The dance practice was performed in a social school of Pontegrande, Catanzaro, in the south of Italy. In detail, subjects took part 3 times/week in dance classes (Monday, Wednesday, and Thursday) and 1 time/week in free dance (Saturday). Subjects did not pay for both dance classes and for free dance. Dance practice, lasted 2 hours, while free dance lasted at least 1 hour and 30 minutes. During the free dance condition, the subjects danced in a club and we allowed them to behave freely without particular restrictions. The subjects were not allowed to drink any alcoholic beverage to not compromise the physical response. Two senior dance-certified instructors conducted and supervised the dance practice during the 6-month training. Subjects' heart rate was continuously recorded beat by beat with a short-range telemetry HR monitor (RS 400, Polar, Electro™, Kempele, Finland) to assess the mean heart rate during all dance practices. Dance classes consisted of different choreographies, which include rhythmic and simple movements typically of Latin dance (Cha Cha Cha, Rumba, Jive, Tango, and Merengue), Caribbean dance (Salsa and Bachata), and folkloristic dance (Tarantella and Pizzica). In all the described dances, the leaders were the men and the followers were the women.

Before (T0) and after the 6 months (T6) of intervention, anthropometric characteristics, thyroid hormones, physical fitness, sexual health, and general cognitive functions were evaluated in each participant. Before the first testing session, participants took part in a familiarization session to become accustomed to the physical fitness tests. All subjects were tested in the morning from 9.00 am to 12 pm.

### 2.3. Anthropometric Characteristics and Body Composition Assessments

Weight and height were measured using a scale and a stadiometer to the nearest 0.1 kg and 0.1 cm, respectively. Body mass index (BMI) was calculated as ratio between weight and square of the height (kg/m^2^). Moreover, for each subject, percent of fat mass (%FM) and fat free mass (FFM) were measured by hand-to-foot bioelectrical impedance method (Tanita BC 601, Tokyo, Japan) with standard clothing (i.e., underwear).

### 2.4. Laboratory Measures of Thyroid Function

The evaluation of thyroid function was performed using a procedure described in detail elsewhere [16]. In summary, blood samples were collected in the morning, after an overnight fast and intra- and interassay coefficients of variation were below 3 and 5%, respectively, for both FT4 and FT3.

### 2.5. Physical Fitness Assessment

To minimize the effects of fatigue, testing stations were organized in the following order: Short Physical Performance Battery (SPPB) [29], 30s Sit-to-Stand test (30CST) [30], Handgrip test (HG) [31], Timed Up-and-Go test (TUG) [32], and 2-Minute step test (ST) [33]. Physical fitness assessment procedure is described in detail elsewhere [16]. In summary, balance was evaluated using a tandem test, gait speed was evaluated using a 4m walking test (no canes or walkers were allowed to be used), and lower body strength was evaluated using the Chair Stand test (CST). The* 30s Sit-to-Stand test (30CST) *was administered using a chair without arms (high chair =43.2). Subjects were asked to fully stand and then returned to the initial seated position for 30 sec consecutively. Incorrectly executed stands were not counted. The participants' hand grip strength was evaluated as left or right according to the dominant hand. To complete the physical fitness assessment, subjects performed TUG and ST tests. For each test (4m walking test, CST, HG, and TUG), the average of two attempts was recorded for data analysis.

### 2.6. Sexual Health Assessment

Subjects' sexual health was evaluated throughout the Changes in Sexual Functioning Questionnaire-short form (CSFQ-14) [34] and the Sexual Attitude Scale (SAS) [35] questionnaires. The CSFQ-14 is a clinical and research instrument identifying five scales of sexual functioning, while the SAS estimates the conservative or the liberal propensity toward sexual expression. Details of these questionnaires are depicted in detail elsewhere [16].

### 2.7. Cognitive Function Assessment

Subjects' general cognitive functions were assessed by using a standardized neuropsychological battery test. The following tests were administrated: Mini Mental State Examination (MMSE) [36] was used to screen cognitive impairment. In addition, the prospective-retrospective memory self-report questionnaire (PRMQ) [37] was used to measure the subjective impairment in daily life in prospective and retrospective memory. It is a self-report questionnaire and it is formed by sixteen items, eight asking about prospective memory (PM) (i.e., the ability to remember to accomplish an intended action at some point in the future such as remembering to take a tablet after lunch) and eight about retrospective memory (RM) failures. Higher scores in the PRMQ indicate a higher incidence of problems in PM and RM. The Geriatric Depression Scale (GDS) [38] was used to evaluate depressive symptoms of both groups.

Subjects' anxiety was assessed by the State-Trait Anxiety Inventory. Form Y1 (STAI-Y1) provides scores for state anxiety and Form Y-2 (STAI-Y2) [39] provides scores for trait anxiety levels. The entire neuropsychological battery lasted around 45 to 60 minutes.

### 2.8. Statistical Analysis

To verify the data distribution, the Kolmogorov-Smirnov test was performed, which revealed that the variables had normal distribution. In this case, the mean and standard deviation for all variables were calculated. At the beginning of the study, differences on body composition between males and females were evaluated with the unpaired* t*-test. For each variable, a 2 (time) X 2 (gender) ANOVA with repeated measures on time factor was conducted to examine main and interaction effects. If significant differences were obtained, a post hoc test was conducted. Statistical significance was set a priori at P ≤ 0.05. All data were analysed using SPSS (version 24.0 for Windows; SPSS Inc., Chicago, IL).

## 3. Results

Twenty-five of the thirty enrolled participants (age: 71.4±5.8 years, 17 females and 8 males) completed the study. In detail, three subjects interrupted the study for new development of disease, 1 for ankle pains, and 1 for sickness of the partner. No subjects had thyroid diseases. Values of thyroid hormones were within normal range (TSH: 0.4-4.0 mIU/L; FT3: 2.3-4.2 pg/mL; FT4: 0.70-1.76 ng/dL): in further detail, mean thyroid-stimulating hormone (TSH) level was 1.4±0.9 mIU/L in females and 1.2±0.7 mIU/mL in males; free triiodothyronine (FT3) was 3.0±0.5 pg/mL in females and 3.2±0.22 pg/mL in males, while free thyroxine (FT4) was 1.4±0.9 ng/dL in females and 1.4±0.5 ng/dL in males.

Subjects' average heart rate was equal to 64% and 58% of their maximum heart rate calculated as 220 minus age in dance classes and free dance, respectively.

Significant differences between males and females for weight, height, and FFM were observed at T0 (Table 1). Significant main effect of time was found for %FM (F_1,23_=42.9, p < 0.01, *η*p^2^ 0.65) and FFM (F_1,23_=28,2, p < 0.01, *η*p^2^ 0.55). Moreover, a significant main effect of gender on weight (F_1,23_=5.1,* p *= 0.03, *η*p^2^ 0.18), height (F_1,23_=32.9,* p* < 0.01; *η*p^2^ 0.59), %FM (F_1,23_=6.57,* p* < 0.02, *η*p^2^ 0.22), and FFM (F_1,23_ =13.0,* p *< 0.01, *η*p^2^ 0.36) was found (Table 1). No significant time x gender interactions on anthropometric variables were observed.

Regarding the physical fitness parameters, HG was significantly higher in males than in females at T0 and a significant main effect of time on CST (F_1,23_=19.6,* p* < 0.01, *η*p^2^ 0.46), GS (F_1,23_=6.48,* p*=0.02, *η*p^2^ 0.22), and TUG (F_1,23_=4.37,* p*=0.048, *η*p^2^ 0.16) was found (Table 2). A significant main effect of gender on HG (F_1,23_=9.26, p = 0.01, *η*p^2^ 0.29) was found (Table 2). No significant time x gender interactions on physical fitness variables were observed. Regarding subjects' sexual health, CSFQ-14 was significantly higher in males than in females at T0 and a significant main effect of gender on CSFQ-14 (F_1,23_=9.87.48,* p*=0.01, *η*p^2^ 0.30) was found (Table 3). No significant main effects of time and time x gender interactions on sexual health variables were observed.

A significant main effect of time on STAI-Y2 (F_1,23_=6.90,* p*=0.02, *η*p^2^ 0.23) and PRMQr was found (F_1,23_=7.31,* p*=0.01, *η*p^2^ 0.24). In detail, trait of anxiety decreased, and perception of retrospective memory increased after training independently of the gender (Table 3). No significant main effects of gender and time x gender interactions on psychological variables were observed.

## 4. Discussion

The present study aimed to investigate whether the 6-month social dance practice could affect subjects' body composition, physical fitness, and sexual and psychological aspects in older experienced dancers. The most pronounced observation from the results of this study is that even if subjects were experienced dancers, body composition, some physical fitness parameters, memory, and mood improved after training. On the other hand, no subjects' sexual health improvement was observed.

Despite the nonsignificant differences on body weight, we found that %FM decreased and FFM increased after intervention in euthyroid subjects. To the best of our knowledge, this is the first study that showed a change in body composition after social dance practice in older subjects. Hopkins [40] found a decreased fat mass and an increased fat free mass after a low-impact aerobic dance in sedentary elderly women. If we compare our results to those reported by Hopkins [40], we might speculate that social dance practice might have positive results on subjects' body composition also in experienced dancers and not only in sedentary women. This positive result might be explained by the exercise intensity and the volume of dance practice. In fact, as described in the results section, the exercise intensity of social dance was moderate and consequently based mostly on the aerobic metabolism which utilizes mostly fat as energy substrate.

Also, we found that both CST and TUG tests improved after dance practice. These two parameters are strongly correlated with the risk of falls. In fact, higher lower body strength and better body agility might result in a decreased risk of falls [41]. On the other hand, our results did not show any differences in balance, strength, and step test. Our results of physical performance might have been influenced by the subjects' physical level before the study. In fact, scientific evidence showed that the practice of 12 weeks of dance had positive results on muscle strength [28] and balance [27] in either beginners or unhealthy individuals. Our subjects were healthy, and they have been practicing social dance for at least 5 years. Therefore, improvement of physical fitness might be lower in subjects with good technical ability compared to the beginners and/or unhealthy subjects. Moreover, the kind of movements performed during the 6-month dance practice might not be enough to improve muscle strength. In fact, in order to improve muscle mass and consequently muscle strength, muscle should be stressed with an overload higher than which it is usually stressed. Therefore, the workload of dance practice might not be enough to increase muscle strength in elderly experienced dancers. Further, the lack of significant difference in subjects' balance might be also explained by the high balance ability of our subjects as indicated by the high score of balance test at the beginning of the study.

In accordance with our previous results [16] and recent scientific literature [42, 43], males have a higher sexual health than females. In our previous study [16], we demonstrated the relationship between physical fitness and sexual health in older adults. In detail, a better physical fitness is correlated with better sexual health in older adults. In our results, CSQF-14 score was lower in females than in males, suggesting a worse sexual behavior, likely due to different sexual concerns in females as compared to males. As previously reported [16], these results might be dependent on the higher conservative attitudes in females related to the typical social and cultural factors of a small town in Southern Italy [16]. In fact, we might speculate that in a small town in Southern Italy, older women are less interested in sexual activities and they are strongly influenced by the catholic tradition.

It would be very interesting to study the differences in sexual health and attitudes between big city and small town or between Southern and Northern Italy. Even if social dance is often danced merely to socialize and for entertainment, it may have also sexual functions. However, our results demonstrated that social dance practice did not influence sexual health and sexual attitude in older experienced dancers. The lack of effect on sexual health might be influenced by the trait of personality. In fact, it has been shown that subjects' personality might differently influence how dance is experienced leading to different effects on sexual health [44].

Further studies are needed to verify the effects of different dance styles or longer dance intervention on sexual health in different decades of life. Regarding the effects of social dance on cognitive and psychological characteristics, memory, and anxiety, a significant main effect of time for trait anxiety was found. Differently from what it was expected, trait of anxiety decreased after dance training, while state of anxiety did not change. State-anxiety is defined as a transitory emotional condition characterized by subjective, consciously perceived feelings of tension and apprehension and by strong autonomic nervous system activity; instead, trait-anxiety is defined as a general tendency to respond with anxiety to perceived threats, and it is a relatively stable characteristic of an individual [45]. We might hypothesize that the practice of dance and in particular social dance might change the relationship with other people, changing also the manner to perceive threats in the environment. Interestingly, we found no significant changes in state-anxiety. We might speculate that elderly dancers were not in a transitory emotional condition during this period.

Regarding prospective (PM) and retrospective (RM) memories, we found significant positive effects of social dance on retrospective memory. PM is the cognitive ability of remembering to execute delayed intentions in the future, whereas RM refers to the recalling of past events, words, or people [39]. The present study shows that the practice of social dance might improve RM. This may be important for the daily serenity of old people who often feel they are worried about forgetting everything and getting old.

The effects of dance on physical fitness, sexual health, and psychological variables might be influenced by the dancing motivation [46]. Scientific literature showed that dance motivation appears to be similar to those identified in other forms of behaviour such as exercise [46].

We are aware that major study limitations were the number of males, lower than females, and the lack of steroid hormones evaluation for all subjects. Moreover, in our study, no control group was analysed making it difficult to discuss the nonsignificant effect of dance on the analysed variables. Further studies are needed in order to better analyse the effects of dance practice, taking into consideration not only the dance style, but also the motivation to dance.

## 5. Conclusion

This study indicates that the benefits gained from social dance intervention could regard multiple domains and these positive effects might be reached not only in beginner, but also in advanced dancers. The social dance practice could decrease the aging process through the practice of an activity that also improves socialization in elderly subjects. There is a strict relationship between active sexuality, preserved cognitive function, and appropriate physical fitness in elderly subjects. However, no effects of 6-month dance practice on sexual health were found in intermediate-level dancers. Future investigations are needed in order to study the effects of different type of dance, subjects' motivation, and longer dance practice on subjects' well-being in different elderly populations.

## Figures and Tables

**Table 1:** Subjects' anthropometric characteristics in pre- (T0) and postdance (T6) practice. Values are represented as mean ± SD.

	Females (n=17)		Males (n=8)		Pooled (n=25)	
	T0	T6	T0	T6	T0	T6
Weight (cm)	67.0 ± 11.0	68.5 ± 11.1	77.1 ± 6.9^a^	76.7 ± 7.2	70.2 ± 10.9	70.5 ± 10.8
BMI (kg/m^2^)	29.2 ± 4.4	30.1 ± 5.8	28.0 ± 2.8	27.9 ± 3.3	28.8 ± 3.9	29.4 ± 5.2
%FM (%)	42.1 ± 8.0	38.5 ± 6.6	33.7 ± 9.3^a^	29.9 ± 8.6	39.4 ± 9.2	35.8 ± 8.2^*∗∗*^
FFM (kg)	21.5 ± 3.9	23.0 ± 4.1	27.7 ± 5.2^b^	30.2 ± 5.4	23.5 ± 5.2	25.3 ± 5.6^*∗∗*^

^*∗∗*^P≤0.01 vs. pre; ^a^P≤0.05 vs. females; and ^b^P≤0.01 vs. females.

**Table 2:** Subjects' physical fitness parameters in pre- (T0) and postdance (T6) practice. Values are represented as mean ± SD.

	Females (n=17)		Males (n=8)		Pooled (n=25)	
	T0	T6	T0	T6	T0	T6
Balance (p.ti)	4.0 ± 0.0	4.0 ± 0.0	4.0 ± 0.0	4.0 ± 0.0	4.0 ± 0.0	4.0 ± 0.0
CST (sec)	7.79 ± 1.58	6.17 ± 1.47	7.71 ± 1.78	6.64 ± 2.03	7.77 ± 1.61	6.31 ± 1.64^*∗∗*^
GS (sec)	4.92 ± 0.96	4.67 ± 0.86	5.26 ± 0.93	4.42 ± 1.04	5.03 ± 0.95	4.59 ± 0.91^*∗*^
SPPB (p.ti)	11.5 ± 0.9	11.6 ± 0.6	11.4 ± 0.7	11.3 ± 0.7	11.4 ± 0.8	11.5 ± 0.65
30CST (n)	22.6 ± 6.9	17.7 ± 5.3	17.6 ± 5.9	17.9 ± 4.1	21.0 ± 6.9	17.8 ± 4.85
TUG (sec)	8.00 ± 1.10	7.50 ± 1.30	8.20 ± 1.20	7.70 ± 1.30	8.07 ± 1.14	7.59 ± 1.28^*∗*^
ST (step)	110.0 ± 35.6	129.3 ± 34.9	126.3 ± 31.5	126.0 ± 33.5	115.3 ± 34.5	128.3 ± 33.8
HG (kg_f_)	21.2 ± 4.2	21.3 ± 4.6	27.4 ± 7.5^a^	29.3 ± 8.7	23.2 ± 6.1	23.8 ± 7.12

CST: Chair Stand test; GS: Gait Speed; SPPB: Short Physical Performance Battery; 30CST: 30s Sit-to-Stand test; TUG: Timed Up-and-Go test; ST: Step Test; and HG: Hand Grip. ^*∗*^P≤0.05 vs. pre; ^*∗∗*^P≤0.01 vs. pre; and ^a^P≤0.05 vs. females.

**Table 3:** Subjects' sexual health and psychological variables in pre- (T0) and postdance (T6) practice. Values are represented as mean ± SD.

	Females (n=17)		Males (n=8)		Pooled (n=25)	
	T0	T6	T0	T6	T0	T6
CSFQ-14 (score)	28.8 ± 11.4	28 ± 10.9	41.8 ± 7.8^b^	41.6 ± 5.9	32.9 ± 12.0	32.5 ± 11.4
SAS (score)	78.5 ± 15.6	78.8 ± 15.4	75.8 ± 13.6	74.0 ± 14.5	77.6 ± 14.8	77.2 ± 14.5
MMSE (score)	25.9 ± 1.76	25.3 ± 2.6	26.6 ± 2.6	27.0 ± 2.5	26.1 ± 2.0	25.8 ± 2.6
GDS (score)	2.2 ± 3.0	2.4 ± 3.5	1.9 ± 2.5	1.6 ± 2.4	2.1 ± 2.8	2.2 ± 3.1
PRMQp (score)	21.3 ± 6.5	19 ± 6.5	18.6 ± 5.4	19 ± 4.9	20.4 ± 6.2	19 ± 5.9
PRMQr (score)	18.0 ± 6.2	16 ± 6.5	19.8 ± 6.8	19 ± 5.9	17.1 ± 6.3	38.1 ± 9.6^*∗*^
STAI-Y1 (score)	36.6 ± 8.7	37 ± 13.9	41.3 ± 1.3	40 ± 8.4	38.1 ± 9.6	37.7 ± 12.3
STAI-Y2 (score)	41.8 ± 8.9	36.1 ± 10.4^*∗*^	40.0 ± 6.1	37.0 ± 6.2	41.2 ± 8.0	36.4 ± 9.2^*∗*^

CSFQ-14: Changes in Sexual Functioning Questionnaire-short form; SAS: Sexual Attitude Scale; MMSE: Mini Mental State Examination; GDS: Geriatric Depression Scale; PRMQp: Prospective Memory; PRMQr: Retrospective Memory; STAI-Y1: State Anxiety Inventory; and STAI-Y2: Trait Anxiety Inventory. ^*∗*^P<0.05 vs. pre; ^b^P≤0.01 vs. females.

## Data Availability

The data used to support the findings of this study are available from the corresponding author upon request.
